# Natural Killer Cells from Malignant Pleural Effusion Are Endowed with a Decidual-Like Proangiogenic Polarization

**DOI:** 10.1155/2018/2438598

**Published:** 2018-03-29

**Authors:** Annalisa Bosi, Silvia Zanellato, Barbara Bassani, Adriana Albini, Alessandra Musco, Maria Cattoni, Matteo Desio, Elisa Nardecchia, Davide Giuseppe D'Urso, Andrea Imperatori, Lorenzo Dominioni, Douglas M. Noonan, Lorenzo Mortara, Antonino Bruno

**Affiliations:** ^1^Vascular Biology and Angiogenesis Laboratory, Scientific and Technology Pole, IRCCS MultiMedica, Milano, Italy; ^2^Immunology and General Pathology Laboratory, Department of Biotechnology and Life Sciences, University of Insubria, Varese, Italy; ^3^Department of Medicine and Surgery, University of Insubria, Varese, Italy

## Abstract

Natural killer (NK) cells are crucial in tumor recognition and eradication, but their activity is impaired in cancer patients, becoming poorly cytotoxic. A particular type of NK cells, from the decidua, has low cytotoxicity and shows proangiogenic functions. We investigated whether NK cells from peripheral blood (PB) and pleural effusions of patients develop decidual-like NK phenotype and whether exposure to IL-2 can restore their killing ability in the presence of pleural fluids. NK cells from pleural effusion of patients with inflammatory conditions (iPE, *n* = 18), primary tumor (ptPE, *n* = 18), and metastatic tumor (tmPE, *n* = 27) acquired the CD56^bright^CD16^−^ phenotype. NK cells from both ptPE and tmPE showed increased expression for the CD49a and CD69 decidual-like (dNK) markers and decreased levels of the CD57 maturation marker. NK from all the PE analyzed showed impaired degranulation capability and reduced perforin release. PE-NK cells efficiently responded to IL-2 stimulation *in vitro*. Addition of TGF*β* or cell-free pleural fluid to IL-2 in the culture medium abrogated NK cell CD107a and IFN*γ* expression even in healthy donors (*n* = 14) NK. We found that tmPE-NK cells produce VEGF and support the formation of capillary-like structures in endothelial cells. Our results suggest that the PE tumor microenvironment can shape NK cell polarization towards a low cytotoxic, decidual-like, highly proangiogenic phenotype and that IL-2 treatment is not sufficient to limit this process.

## 1. Introduction

Natural killer (NK) cells are lymphoid cells of the innate immune system included in the recent redefined family of innate lymphoid cells (ILC) as type 1 ILC [1, 2] and represent about 10–20% of total lymphocytes in human peripheral blood (PB) [3]. The new ILC classification reconsiders the innate cells and their specialized functions based on transcription factor and cytokine expression, underlying some similarities with T cell functions [2]. NK cells have both effector and regulatory activities against tumor and virus-infected cells through cytotoxicity and cytokine release. They are characterized by cytoplasmic granules and exert their cytotoxicity by releasing perforin and granzyme molecules, able to trigger target cell death by apoptosis. NK cells also play a crucial role in the production of immunoregulatory cytokines and chemokines [3–8]. These soluble factors impact on recruitment and function of other hematopoietic cells, placing NK cells as orchestrators in the crosstalk between innate and adaptive immunity [9, 10]. NK cells are characterized by a wide array of activating and inhibitory receptors that are finely tuned according to their functions and the hosting microenvironment [2].

Based on the expression density of the CD56 and CD16 surface antigens, two predominant morphologically and functionally different NK cell subsets have been identified in peripheral blood (PB-NK) [8, 11]. Approximately 95% of PB-NK cells are characterized by a CD56^dim^CD16^+^ phenotype, can release cytolytic granules containing perforin and granzyme, and exert cytotoxicity [12]. The other subpopulation in PB-NK (about 5%) shows a CD56^bright^CD16^−^ phenotype: these cells are poorly cytotoxic, yet they are able to secrete high levels of cytokines [8, 11]. CD56^bright^CD16^−^ NK cells are mostly present in secondary lymphoid organs, and recent studies have attributed a new immunomodulatory role in the context of chronic inflammatory conditions (autoimmune disease), the ability to inhibit proliferation of autologous CD4^+^ T cells [13, 14].

The CD56^bright^CD16^−^ NK cell subset has been supposed to represent preterminally differentiated NK cells, capable of cell proliferation and cytokine production. Following exposure to interleukin-2 (IL-2), IL-12, and/or IL-15, these cells can differentiate into the phenotype CD56^dim^CD16^+^, showing higher levels of perforin and granzymes [5, 15] and cytotoxicity.

During the first trimester of pregnancy, a third NK cell subset has been identified within the decidua, termed decidual or uterine NK (dNK) cell, that comprises 30–50% of all lymphoid cells in the decidual tissue. dNK cells are characterized by a CD56^superbright^CD16^−^ phenotype, along with the expression of specific surface markers, such as CD9 (a tetraspanin protein family that plays a role in cell adhesion and cell motility), CD49a (an integrin alpha subunit that binds collagen and laminin), and CD69, an early activation molecule [7, 16, 17]. dNK cells strongly support angiogenesis [7, 16, 17] by releasing vascular endothelial growth factor (VEGF) [18, 19], placental growth factor (PlGF), and IL-8 [16] and are responsible for spiral artery formation [20]. dNK cell supernatants show a potent proangiogenic action *in vitro* and *in vivo* and are able to significantly increase tumor growth and angiogenesis [16]. dNK cells represent a clear example of NK cell plasticity that, in a peculiar physiological environment, can be switched from killers to builders [21]. It is now clear that the tumor microenvironment employs multiple mechanisms to switch off the antitumor functions of immune cells and can alter and polarize the innate cell compartment (e.g., macrophages, neutrophils, and dendritic cells) or stromal cells (cancer-associated fibroblasts) for growth and dissemination [6, 21–24]. It has been shown that NK cells could be conditioned by a solid tumor microenvironment to become a protumor immune cell subset with low cytotoxic capacity and acquisition of dNK-like features [6, 21, 25].

By translating this concept in the tumor context, we were the first in demonstrating that in non-small-cell lung cancer (NSCLC) patients, tumor-infiltrating NK cells (TINKs) are enriched in the CD56^bright^CD16^−^ NK cell subset and acquire proangiogenic features. NSCLC TINKs and tumor-associated peripheral blood NK cells (TANKs) can release VEGF, PlGF, and IL-8, and functional assays showed that conditioned media from TANKs and TINKs induce capillary-like structure formation by human umbilical vein endothelial cells (HUVEC) *in vitro*. We identified TGF*β*, a potent immunosuppressor factor within the tumor microenvironment, as a master proangiogenic switch in NK cells [21, 25]. However, other factors within the tumor microenvironment such as adenosine, hypoxia, and PGE2 could also be responsible or cooperate with TGF*β*, resulting in the conversion of antitumor NK cells into protumor, proangiogenic decidual-like cells [21, 26, 27].

In a cohort of 25 patients, it has been found that NK cells in the pleural effusions (PE-NK) of patients with primary or metastatic tumors of different origins display an expansion of the CD56^bright^ NK cell subset and can release large amounts of IFN*γ* and TNF*α*, following PMA/Ionomycin stimulation [28]. Here, we studied in a larger cohort of patients (*n* = 78) to investigate whether NK cells isolated from pleural effusion of malignant primary (ptPE) or metastatic tumors (tmPE), apart from impaired killing capability, can acquire proangiogenic phenotype and function. We also assessed whether NK cells from pleural effusion of tumors of different origin were able to respond to IL-2 short-time treatment, both when directly isolated from PE fluids or when polarized with PE fluids *in vitro*. We also include PE derived from patients with inflammatory nononcologic condition, to dissect whether potential proangiogenic NK cell polarization can be a distinctive tumor feature.

## 2. Material and Methods

### 2.1. Patient Selection

Subjects included in this study were patients with pleural effusions (PE) induced by either inflammatory disease, primary, or metastatic tumors of different origin (*n* = 63, 36 men and 27 women, 18 with inflammatory pleural effusions, 18 with pleural effusion caused by primary tumors, and 27 with pleural effusion caused by metastatic tumors) and healthy controls (*n* = 14, 8 men and 6 women) (Table 1) under a local ethics committee approval, and informed consent was obtained for each donor. Patients and healthy donors were from 45 to 91 years old. Samples analysed included peripheral blood of healthy subjects (hPB) and patients with inflammatory conditions (iPB), primary tumors (ptPB), or tumor metastasis (tmPB) and the respective pleural effusions: iPE, ptPE, and tmPE.

Patients with diabetes, human immunodeficiency virus (HIV)/hepatitis C virus (HCV)/hepatitis B virus (HBV) infection, overt chronic inflammatory conditions, previously treated with chemotherapy or radiotherapy, or those iatrogenically immunosuppressed or having undergone myeloablative therapies, were excluded.

### 2.2. NK Cell Phenotype Characterization

Total mononuclear cell suspension derived from peripheral blood and pleural effusion samples was obtained by ficoll hystopaque (Lonza, Basel, Switzerland) gradient stratification [25]. To identify NK cell subsets, cells obtained were subsequently stained with monoclonal antibodies against surface markers (CD14-PE and CD45-FITC, CD3-PerCP, CD56-APC, CD16-FITC, CD9-PE, CD49a-PE, CD57-PE, CD69-PE, NKp30-PE, NKG2D-PE, and NKG2A-PE) all purchased from Miltenyi Biotec (Auburn, CA) and analysed by a Becton Dickinson (BD) FACSCanto II flow cytometer (Becton Dickinson, CA). Briefly, after physical parameter analysis (FSC/SSC), CD45^+^CD14^−^ lymphocytes were gated and assessed for NK markers. NK cells were gated on CD45^+^CD3^−^CD56^+^ total lymphocytes.

### 2.3. Evaluation of NK Cell Cytokine Production

NK cells were subjected to intracellular cytokine-staining assay following 1 h incubation with monensin (2 mM, BD) or to evaluate the production of IFN*γ*, after an overnight stimulation with PMA (10 ng/ml, Sigma-Aldrich, Milan, Italy) and Ionomycin (500 ng/ml, Sigma-Aldrich) plus monensin (2 mM, BD). Briefly, following staining with anti-human mAbs CD3-PerCP, CD56-APC, and CD16-FITC (Miltenyi Biotec), cells were permeabilized and fixed using the Cytofix/Cytoperm fixation kit (BD), according to the manufacturer's instructions and finally stained with different anticytokine PE-conjugated mAbs (VEGF, SDF-1, perforin, osteopontin, IL-8, or IFN*γ*, all from Miltenyi Biotec).

### 2.4. Detection of NK Cell Degranulation Capacity

The NK cell degranulation activity assay was performed on total mononuclear cells from PB and PE after *in vitro* 4 h incubation with the K562 tumor cell line human target (chronic myelogenous leukemia, ECACC, Sigma-Aldrich) in the presence of conjugated anti-CD107a mAb (Miltenyi Biotec) and monensin (2 mM, BD) at a NK: target cell ratio of 1 : 1 [29]. Cells were then stained with PerCP-conjugated anti-CD3 and APC-conjugated anti-CD56 mAbs (Miltenyi Biotec). This assay was performed on fresh PB of healthy donors as well as PB and PE of the patients after a 3-day cell culture with IL-2 (10 ng/ml), IL-2 plus TGF*β* (10 ng/ml) (Miltenyi Biotec), and IL-2 in a medium containing 33% of iPE, ptPE, or tmPE cell-free supernatant. TGF*β* was used as a positive control for NK cell polarization and induction of anergy. FACS data was obtained with a BD FACSCanto II.

### 2.5. Evaluation of Tube Formation by Human Umbilical Vein Endothelial Cells

NK cells were purified from peripheral blood and pleural effusion samples using CD3 MicroBeads (Miltenyi Biotec): CD3^+^ cells were depleted, and the CD3^−^ fraction was purified using CD56 MicroBeads (Miltenyi Biotec) to isolate CD56^+^ NK cells. Purified NK cells were incubated 6 hrs in serum-free RPMI 1640 (Sigma-Aldrich) containing 1% glutamine (Euroclone, Milan, Italy) and 1% penicillin/streptomycin (Euroclone) at 37°C in 5% CO_2_. Supernatants were collected, depleted of cell debris and residual cells by centrifugation and concentrated with Concentricon devices (Millipore, Temecula, CA) with a 5 kDa membrane pore cut-off [25]. The ability of the NK cell-derived supernatants to induce formation of capillary-like networks of human umbilical vein endothelial cells (HUVEC) was performed by the matrigel morphogenesis assay as previously described [25]. HUVECs (15 × 10^3^ cells/well of a 96-well plate) were resuspended in 0.2 ml of a medium containing NK cell supernatants (protein concentration: 30 *μ*g/*μ*l) obtained as above and transferred to the matrigel-coated (10 mg/ml) wells and incubated for 6 hrs at 37°C, 5% CO_2_. Negative control (CTRL−) consisted in FBS-free Endothelial Basal Medium-2 (EBM, Lonza); positive controls (CTRL+) consisted in Endothelial Growth Medium-2 (EGM-2, Lonza), supplemented with bullet-kit (VEGF, FGF1, FGF2, insulin, hydrocortisone, and heparin; Lonza) containing 10% of FBS. The formation of capillary-like structures was documented with an inverted microscope (Zeiss), and angiogenesis was evaluated using the ImageJ software Angiogenesis Analyzer Tool (https://imagej.nih.gov/ij/).

### 2.6. Statistical Analyses

Flow cytometric analyses were performed using BD FACSCanto II and the BD FACSDiva (v6.1.2) software. Statistical analyses were performed using the GraphPad Prism 7 software (San Diego, CA). One-way ANOVA with uncorrected Fisher's LSD posttest was used to evaluate the statistical significance.

## 3. Results

### 3.1. The CD56^bright^CD16^−^ NK Cell Subset Predominates in Metastatic Pleural Effusions

**Figure 1:**
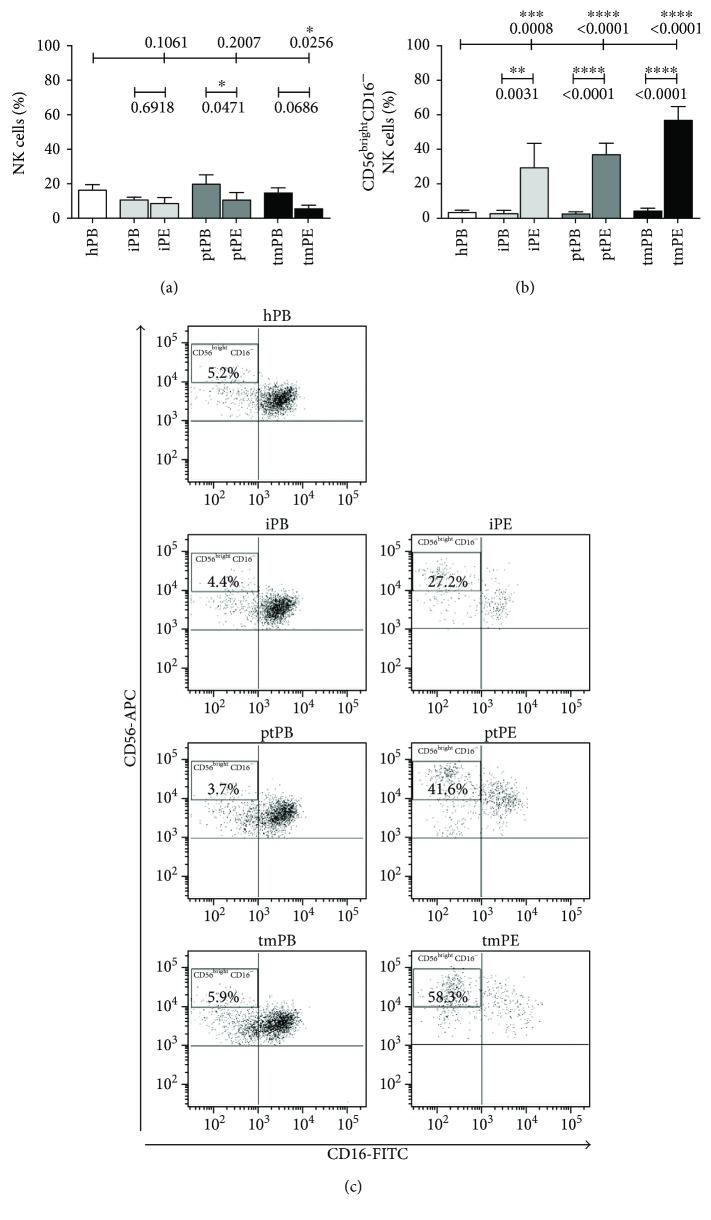
NK cell subset distribution in pleural effusion samples and controls. Flow cytometric analysis showed the percentage of NK cells in all samples from healthy controls (hPB), peripheral blood (iPB) and pleural effusion (iPE) from patients with inflammatory disease, peripheral blood (ptPB) and pleural effusion (ptPE) from patients with primary tumor, and peripheral blood (tmPB) and pleural effusion (tmPE) from patients with tumor metastasis. (a). CD56^bright^CD16^−^ cell subset predominated in all the PE samples with a percentage ranging from 35% in iPE, to 40% in ptPE, and to 60% in tmPE. On the contrary, in PB of pleural effusion patients and healthy controls, the percentages of CD56^bright^CD16^−^ NK cells were approximately 5% (b). Representative dot plots of healthy donors and patients with inflammatory, primary, and metastatic tumor PE are shown, respectively (c). Data are shown as mean ± SEM of 46 samples; ^∗^ *p* < 0.05, ^∗∗^ *p* < 0.01, ^∗∗∗^ *p* < 0.001, and ^∗∗∗∗^ *p* < 0.0001 (*p* values are shown).

Flow cytometric analysis revealed very similar percentages of total NK cells between all PB and PE samples and healthy controls (approximately 10% to 20% of total leukocytes; Figure 1(a)) (Table 1). CD56^bright^CD16^−^ cells were the predominant NK cell subset in all the PE fluids (iPE≈35%; ptPE≈40%; tmPE≈60%), whereas in PB of pleural effusions patients and healthy donors, the percentages of CD56^bright^CD16^−^ NK cells are about 5% (Figure 1(b)). Representative dot plots of patients with inflammatory and primary and metastatic tumor PE show the distribution of CD56^bright^CD16^−^ NK cell subset in PB and PE samples (Figure 1(c)). NK cell activation is the result of a fine-tuned balance between signals transduced by activating receptors, including natural cytotoxic receptors (NKp30, NKp44, NKp46, and NKG2D) and inhibitory receptors. We observed no expression of NKp44 and no modulation NKp46 expression in both PB and PE of all samples (data not shown), as previously reported [30]. We observed downregulation of NKp30 surface receptor and upregulation of the NKG2A inhibitory receptor, although not statistically significant, in all NK cell PE compartments as compared to the PB-NK cells (Supplemental Figure 1).

### 3.2. NK Cells from tmPE Show a Decidual-Like Phenotype and an Alternative Activatory State

**Figure 2:**
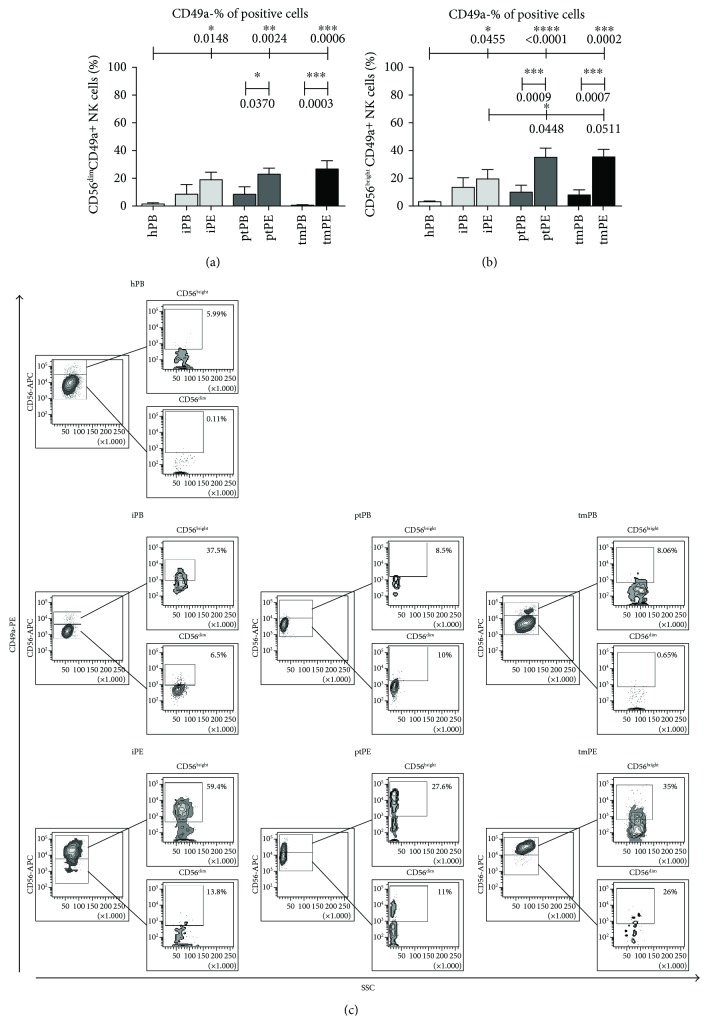
CD49-decidual marker expression on NK cell subsets. Flow cytometric analysis demonstrated that in NK cells from patients with pleural effusions, CD49a expression was increased in the CD56^bright^ subset from pleural effusion (significantly in ptPE and in tmPE) as compared to PB-NK cell samples (a, b). Representative dot plots for CD49a expression on CD56^bright^ and CD56^dim^ NK subsets in healthy donors and patients with inflammatory, primary, and metastatic tumor PE are shown, respectively (c). Data are shown as mean ± SEM of 39 samples; ^∗^ *p* < 0.05, ^∗∗^ *p* < 0.01, ^∗∗∗^ *p* < 0.001, and ^∗∗∗∗^ *p* < 0.0001 (*p* values are shown).

**Figure 3:**
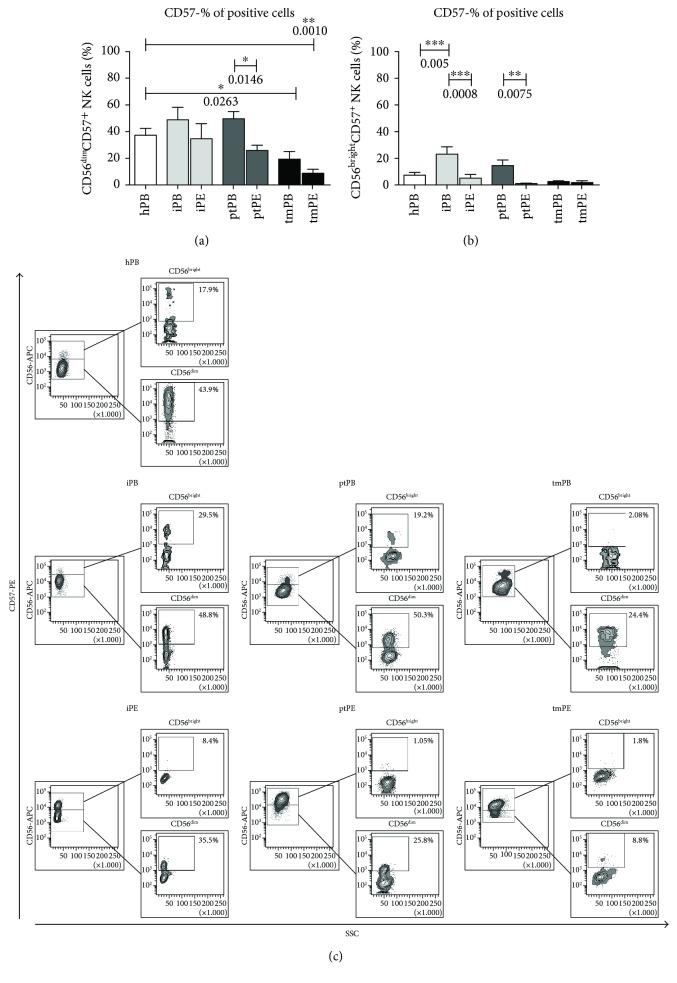
CD57 expression on NK cell subsets. Flow cytometric analysis on NK cell subsets from healthy individuals (hPB), peripheral blood (iPB) and pleural effusion (iPE) from patients with inflammatory disease, peripheral blood (ptPB) and pleural effusion (ptPE) from patients with primary tumor and peripheral blood (tmPB), and pleural effusion (tmPE) from patients with tumor metastasis revealed a decrease percentage of mature NK cells correlated to the downregulation of CD57 marker in PE samples as compared with PB and healthy donors. The percent of CD57 expression was even lower in the CD56^bright^ NK cell subsets, confirming the acquisition of a more immature phenotype (a, b). Representative dot plots of CD57 distribution in CD56^bright^ and CD56^dim^ NK cell subsets (c) in healthy donors and patients with inflammatory, primary, and metastatic tumor PE are shown, respectively. Data are shown as mean ± SEM 39 samples were analyzed; ^∗^ *p* < 0.05, ^∗∗^ *p* < 0.01, and ^∗∗∗^ *p* < 0.001 (*p* values are shown).

**Figure 4:**
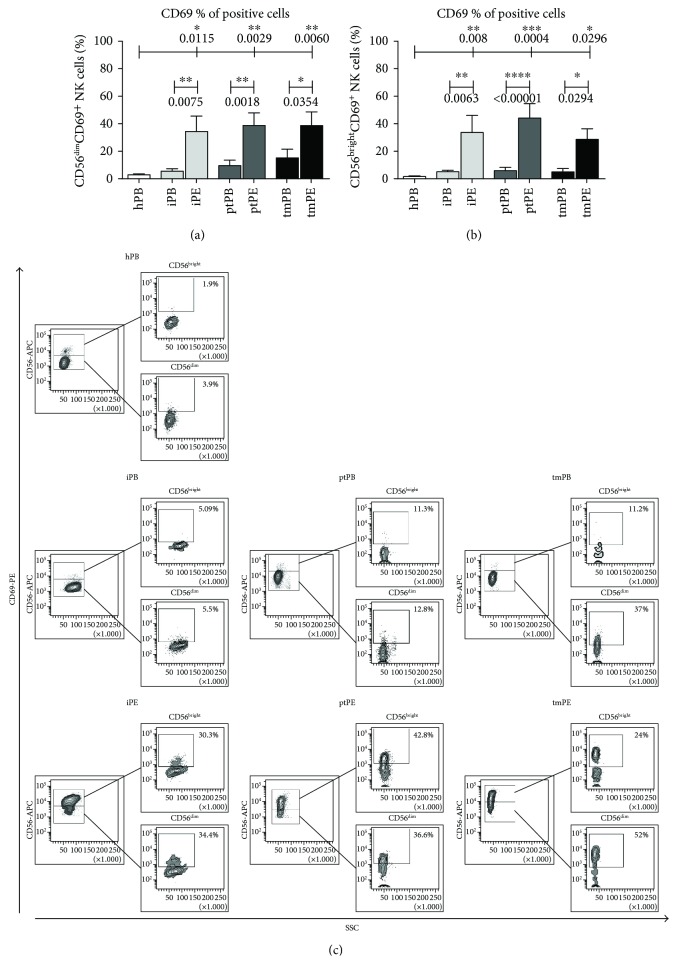
CD69 expression on NK cell subsets. Flow cytometric analysis on NK cell subsets of healthy individuals (hPB), peripheral blood (iPB) and pleural effusion (iPE) from patients with inflammatory disease, peripheral blood (ptPB) and pleural effusion (ptPE) from patients with primary tumor, and peripheral blood (tmPB) and pleural effusion (tmPE) from patients with tumor metastasis revealed upregulation of CD69, an activating and decidual NK marker, in PE samples compared to autologous and healthy control PB-NK cells (a, b). CD69 increased expression was statistically significant in both CD56^bright^ and CD56^dim^ NK cell subsets with higher percentage in CD56^bright^. Representative dot plots of CD69 (c) distribution in CD56^bright^ and CD56^dim^ NK cell subsets in healthy donors and patients with inflammatory, primary, and metastatic tumor PE are shown, respectively. Data are shown as mean ± SEM of 39 samples; ^∗^ *p* < 0.05, ^∗∗^ *p* < 0.01, ^∗∗∗^ *p* < 0.001, and ^∗∗∗∗^ *p* < 0.0001 (*p* values are shown).

We investigated the expression of dNK surface marker expression (CD9 and CD49a) on PE-NK cells. We did not find any differences in CD9^+^ expression on PE-NK cells as a percent of positive cells (Supplemental Figure 1). The proportion of CD49a^+^ NK cells found in pleural effusion was significantly higher as compared to healthy PB-NK cell samples both in the CD56^dim^ and CD56^bright^ subsets and to autologous blood in the patients with tumors (Figures 2(a)–2(c), Supplemental Figure 2). These data suggest an acquisition of a partial decidual-like phenotype. CD57, a marker identifying terminally differentiated cytotoxic mature NK cells with decreased sensitivity to cytokines and a reduced replicative ability [31], was decreased in all PE analyzed as compared to autologous PB samples, which were statistically significant differences for ptPE (CD56^dim^ subset and CD56^bright^ subset) and iPE (CD56^bright^ subset) (Figures 3(a)–3(c), Supplemental Figures 3(a) and 3(b)). PE-NK cells showed a statistically significant upregulation of the CD69 activation and decidual marker [32] in both CD56^dim^ and CD56^bright^ NK subsets compared to autologous and healthy control PB-NK cells (Figures 4(a)–4(c), Supplemental Figures 3(c) and 3(d)). In the context of tumor microenvironment conditions, PE-NK cells are partially activated and not completely inhibited.

### 3.3. Patients with PE Show Impaired NK Cell Degranulation Abilities

**Figure 5:**
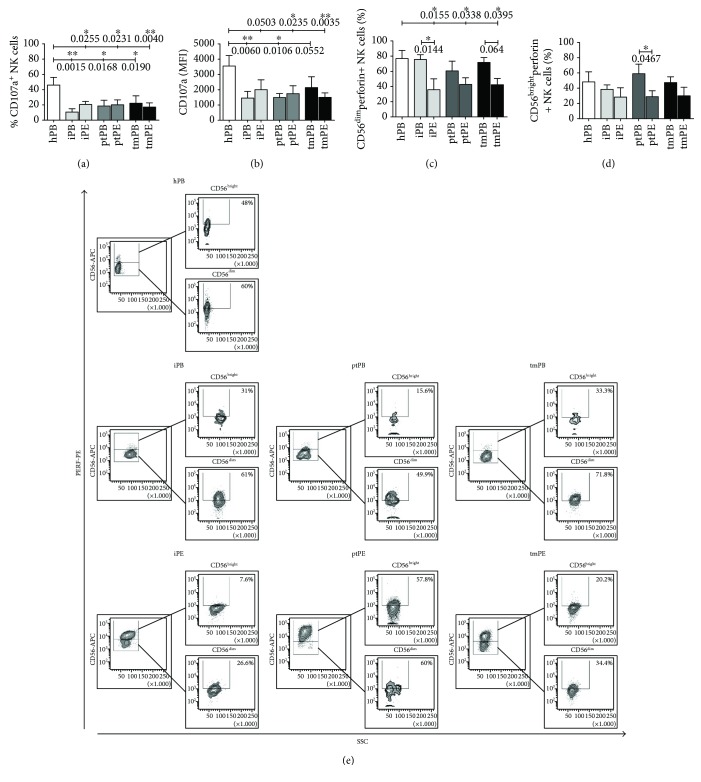
Modulation of degranulation (CD107a) and perforin secretion in NK cell subsets. Flow cytometric analysis for CD107a surface marker showed a downregulation of CD107a expression on PB-NK and PE-NK cells in terms of percentage of positive cells (a) and mean intensity of fluorescence (MFI) (b) that correlated with a significant decrease of the percentage of perforin^+^ NK cells, both in CD56^dim^ and CD56^bright^ subsets in iPE and ptPE (c, d) after exposure to target K562 cells. Representative dot plots of perforin expression in healthy donors and patients with inflammatory, primary, and metastatic tumor PE are shown, respectively (e). Data are shown as mean ± SEM of 34 samples; ^∗^ *p* < 0.05 and ^∗∗^ *p* < 0.01 (*p* values are shown).

NK cells play a relevant role in immunosurveillance, recognizing and killing tumor cells through perforin and granzyme exocytosis and by engagement of death receptors. We evaluated NK cell degranulation potential (using the CD107a surface expression as a marker) and analysed perforin content ex vivo in both PB- and PE-NK cells following 4 hours of incubation with the K562 tumor cell line. The degranulation efficiency was significantly decreased in PE-NK cells (Figures 5(a) and 5(b)) as compared to those from healthy PB. We found that lower degranulation capabilities correlated with a decrease of the percentage of perforin^+^ NK cells in iPE and ptPE (Supplemental Figure 4) as compared to autologous PB-NK, which became significant in iPE, tmPE (CD56^dim^ subset), and ptPE (CD56^bright^ subset) (Figures 5(c)–5(e)). The MFI data indicate that CD56^dim^CD16^+^ NK cells from all patient samples, including PB-NK cells, display lower amounts of perforin when compared to healthy PB controls (Supplemental Figure 4B). These data suggest alterations in NK cell degranulation function also at the systemic level.

### 3.4. PE-NK Cells Are Activated by IL-2 Treatment but Their Degranulation Activity and IFN*γ* Release Are Inhibited by TGF*β* or Autologous PE Fluids

**Figure 6:**
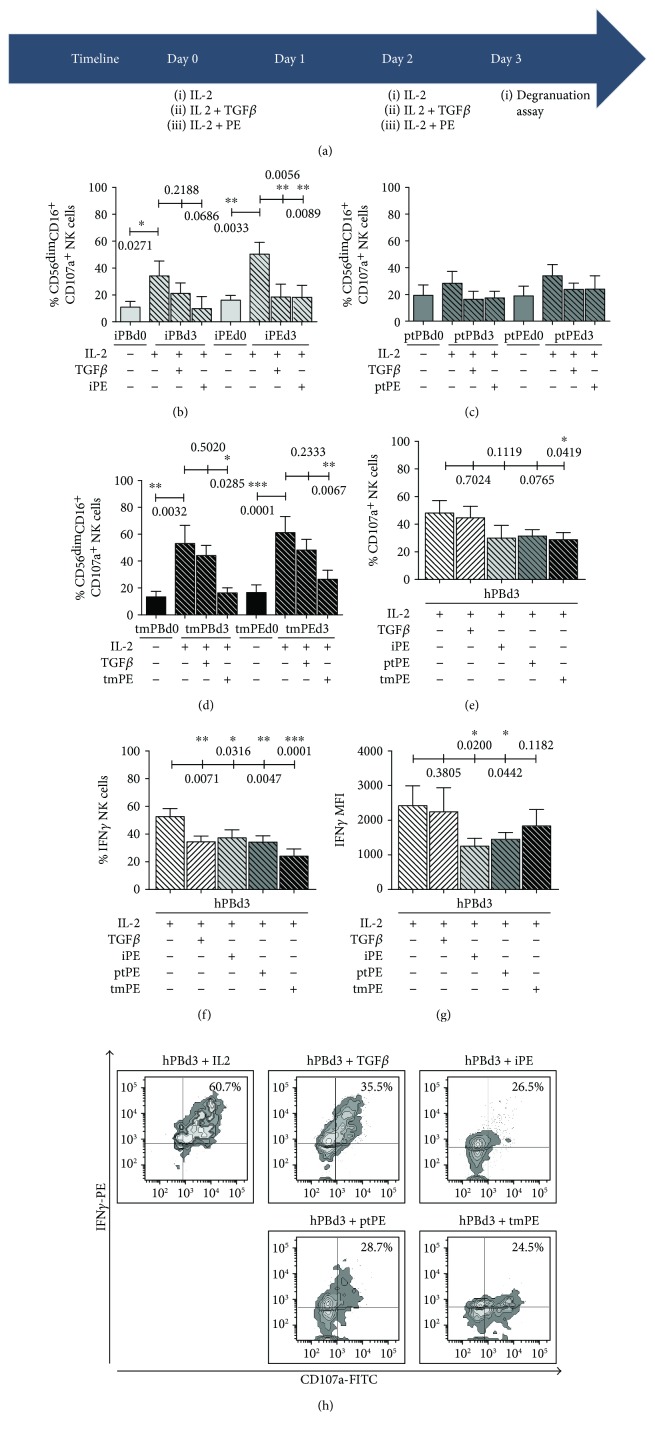
Induction of NK cell anergy by TGF*β* or PE fluids in the presence of IL-2. IL-2, TGF*β*, or autologous PE fluid treatment of NK cells, both from autologous or healthy control-derived NK cells, was performed every 2 days starting from day 0 (D0) for three days as shown by the timeline (a). At day 3 (D3) following coculture with target K562 cells, CD107a modulation was detected by flow cytometry. After IL-2 exposure, PB-NK and PE-NK from patients with PE showed an enhanced cytotoxic potential associated with an increased expression of CD107a. The addition of TGF*β* or inflammatory or metastatic PE supernatants strongly inhibited the IL-2-induced stimulation, inducing a downregulation of CD107a surface marker (b–d). Data are shown as mean ± SEM of 38 samples; ^∗^ *p* < 0.05, ^∗∗^ *p* < 0.01, and ^∗∗∗^ *p* < 0.001 (*p* values are shown). These results were confirmed by polarizing healthy control-derived NK cells with heterologous PE fluids (e–h). The addition of TGF*β*, iPE, ptPE, and tmPE fluids significantly inhibited the percentage of cells expressing CD107a and IFN*γ* and reduced the IFN*γ* MFI (f, g). Representative dot plots of CD107a^+^IFN*γ* ^+^ expression in healthy donors treated with IL-2 and with or without TGF*β*, iPE, ptPE, and tmPE fluids (h) after exposure to target K562 cells. Data are shown as mean ± SEM of 12 samples; ^∗^ *p* < 0.05, ^∗∗^ *p* < 0.01, and ^∗∗∗^ *p* < 0.001 (*p* values are shown).

As previously reported, [30] a short *in vitro* IL-2 stimulation resulted in an impressive cytotoxic NK cell activity from malignant pleural effusion patients (Figure 6). We therefore used IL-2 stimulation, in combination with TGF*β* or PE supernatants, to unveil whether this short 3-day *in vitro* stimulation (Figure 6) could still activate the cytolytic potential of PB-NK and PE-NK cells, which would be more relevant to the clinical situation. This experimental schedule was performed both on autologous PE-derived NKs, as well as by polarizing age-matched healthy control-derived NK cells, with iPE, ptPE, and tmPE liquids. TGF*β* was used as a positive control for NK cell polarization and induction of anergy.

In both PB- and PE-NK from patients, the cytotoxicity of NK cells increased significantly in the presence of IL-2 in the culture medium (Figures 6(a)–6(d)). These data may suggest that the PB-NK and PE-NK of patients are not fully inhibited. During the 3 days of conditioning, the addition of TGF*β* partially or both inflammatory and metastatic PE supernatants strongly inhibited the IL-2-induced stimulation (Figures 6(b)–6(d)). In the IL-2 treatment in the presence of TGF*β* or PE fluids, even healthy donor CD107a^+^IFN*γ* ^+^ NK cells were substantially and significantly repressed (Figures 6(e)–6(h)). These data clearly support the concept that PE fluids contain factors that inhibit the cytotoxic potential of NK cells.

### 3.5. VEGF-Producing NK Cells Are Increased in Metastatic Pleural Effusions

**Figure 7:**
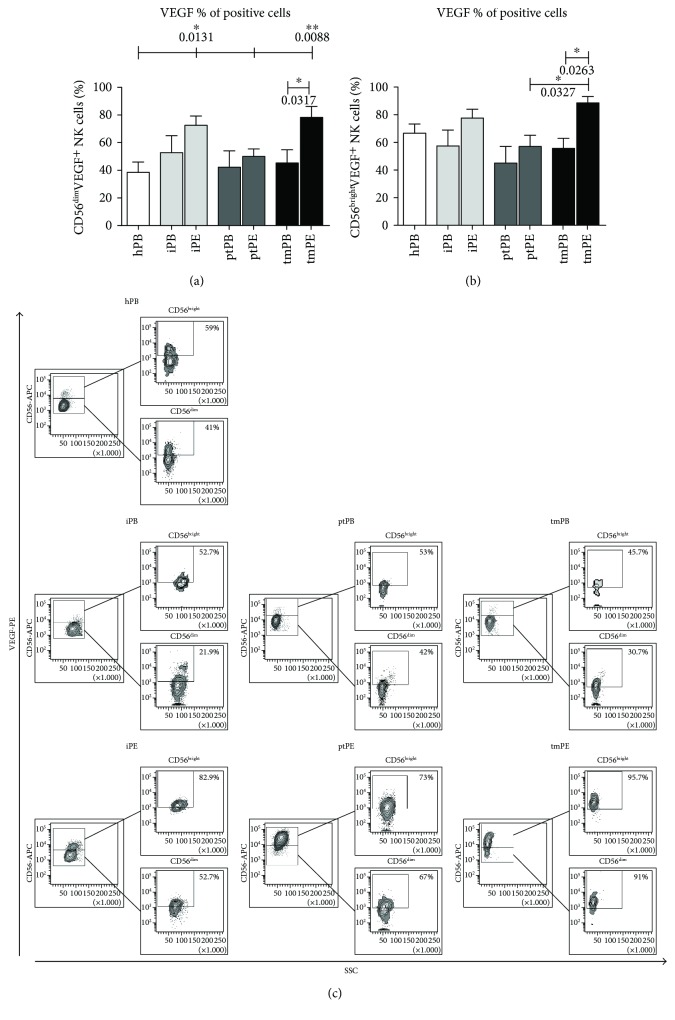
VEGF production by NK cell subsets. Flow cytometric analysis on CD56^bright^ and CD56^dim^ NK cell subsets from healthy individuals (hPB), peripheral blood (iPB) and pleural effusion (iPE) from patients with inflammatory disease, peripheral blood (ptPB) and pleural effusion (ptPE) from patients with primary tumor, and peripheral blood (tmPB) and pleural effusion (tmPE) from patients with tumor metastasis revealed an increased production of VEGF in PE samples as compared with PB and healthy donors (a-b). Representative dot plots of VEGF production by CD56^bright^ and CD56^dim^ NK cell subsets from healthy donors and patients with inflammatory, primary, and metastatic tumor PE are shown, respectively (b). Data are shown as mean ± SEM of 34 samples; ^∗^ *p* < 0.05 and ^∗∗^ *p* < 0.01 (*p* values are shown).

We then focused our attention on NK cell cytokine production from both PB-NK and PE-NK cells without any stimulation in CD56^bright^ and CD56^dim^ NK cells. We tested the production of VEGF, SDF-1, osteopontin, and IL-8. Our data show that NK cells derived from iPE and tmPE produce the proangiogenic cytokine VEGF, in a statistically significant manner, as compared to healthy donor PB-NK cells (Figure 7). In contrast, the production of SDF-1, osteopontin, and IL-8 by NK cells did not display any differences between PB-NK and PE-NK subsets (data not shown).

### 3.6. Supernatants from tmPE-NK Support Angiogenesis *In Vitro*

**Figure 8:**
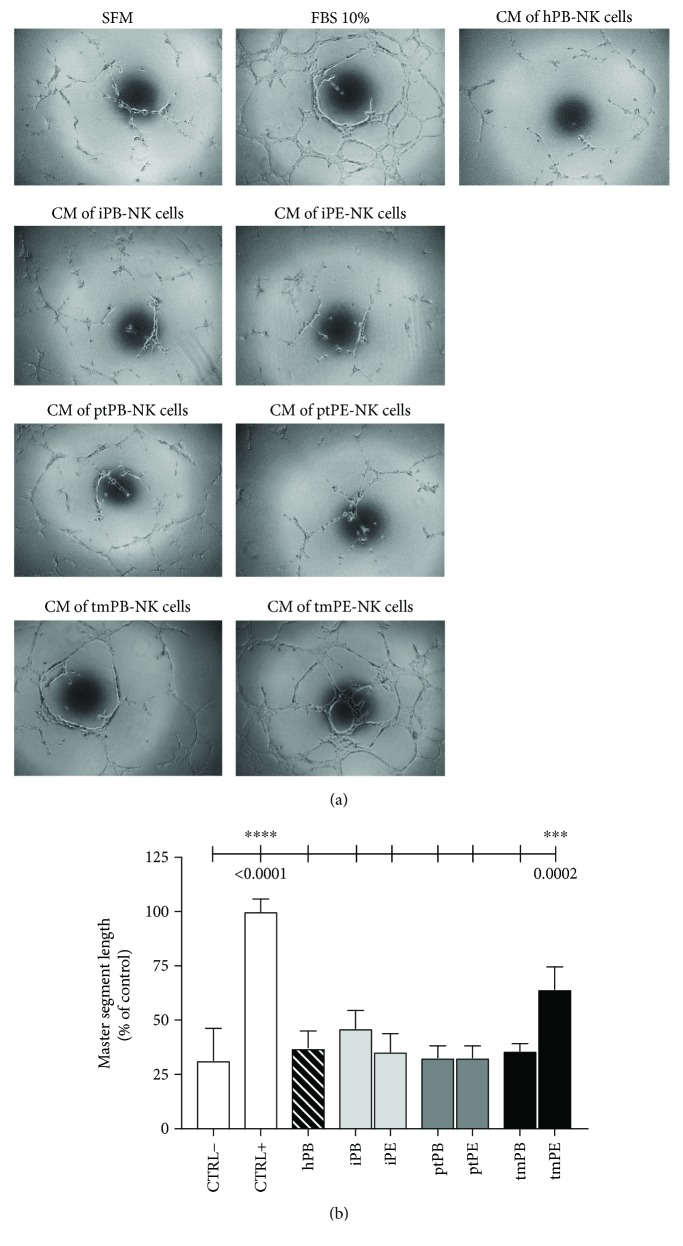
Evaluation of NK cell-derived supernatants' ability to induce morphogenesis on endothelial cells. Following 6 h exposition with NK cell-derived supernatants, tmPE-NK samples showed a significantly higher ability to induce the formation of capillary-like structures on HUVECs seeded on matrigel-precoated plates compared with samples derived from iPE-NK or ptPE-NK cells. Negative control (CTRL−) consisted in FBS-free Endothelial Basal Medium-2 (EBM, Lonza); positive controls (CTRL+) consisted in Endothelial Growth Medium-2 (EGM-2, Lonza), supplemented with bullet-kit (VEGF, FGF1, FGF2, insulin, hydrocortisone, and heparin; Lonza) containing 10% of FBS. Microphotographs (a) were taken at 10x magnification, and master segment lengths (b) were quantified by the Angiogenesis Analyzer ImageJ tool kit. Data are shown as mean ± SEM; ^∗∗∗^ *p* < 0.001 and ^∗∗∗∗^ *p* < 0.0001 (*p* values are shown).

To assess the ability of NK cells to promote angiogenesis, we collected supernatants (SN) from purified NK cells in all samples (inflammatory, primary, and metastatic PB/PE and PB of healthy donors). These were tested on human umbilical vein endothelial cells (HUVECs) using the morphogenesis *in vitro* assay [25]. SN from tmPE-NK cells induce a significantly greater HUVEC morphogenesis compared with SN derived from iPE-NK or ptPE-NK cells (Figure 8). No differences were observed between negative control (serum-free medium, SFM) and SN from PB-NK cells, suggesting that these samples did not exert any proangiogenic actions.

## 4. Discussion

Malignant pleural effusions represent a severe clinical condition in approximately 50% of metastatic cancers and in about 30% of malignant mesothelioma patients, for which there is no effective treatment but only palliative care [33–35]. The median survival of patients with metastatic neoplasia and pleural effusion is three months [33–35]. Although it is known that some specific cancers such as lung, breast, and ovarian preferentially metastasize to the pleural compartment [36–39], the mechanisms are still unknown. It has been shown that inflammatory conditions in the pleura are associated with a supportive microenvironment for the growth of cancer cells [40] with a specific recruitment and accumulation of monocytes/macrophages, dendritic cells, and lymphocytes [41], while only limited information exists on NK cells in malignant pleural effusions.

The concept of “escape from immune surveillance” by which the immune system could be subverted in the context of the tumor microenvironment [42] is the result of the balance between intratumor cytotoxic immune cells and immunosuppressive cells that can be used to predict the outcome of various cancers, such as early-stage colorectal cancer, gastrointestinal cancer, pulmonary adenocarcinoma, and breast cancer [43, 44]. During tumor progression, antitumor effector functions of immune components undergo alterations that lead to inhibition of protective effector functions and acquisition of protumor features [45]. Several studies have found that NK cells are poorly cytotoxic in different cancer types, in particular NSCLC, as reviewed in [21]. In malignant PE, there was an expansion of poorly cytotoxic NK cells associated with an enrichment of CD56^bright^CD16^−^ NK cell subset [30]. A similar pattern of phenotype has been observed in NK cells infiltrating breast cancer [46], colorectal cancer [47], and NSCLC [25, 48]. Conversely, normal lung tissues are moderately rich in NK cells [25, 49] and it was found that the predominant subset in normal parenchyma is the with CD56^dim^CD16^+^ NK cell phenotype [14, 25, 48].

NK cells isolated from different types of PE are characterized by an expansion of CD56^bright^CD16^−^ NK cell subset, compared to autologous PB-NK cells or PB-NK cells from healthy donors, in which CD56^dim^CD16^+^ cytotoxic NK cells prevail. No alteration in the number of total NK cells has been found in PE fluids of different tumors, while differences in NK subset distribution have been observed, with a predominance of poorly cytotoxic NK cells [50]. We found that NK cells in PE from tumors of different origins show lower levels of CD107a expression when exposed to classic target cells and are related to impaired cytotoxicity [51]. These data are in accordance with those demonstrating defective NK cell cytotoxicity in NSCLC, colorectal, breast, and ovarian cancers [14, 25, 46–48]. We hypothesized that the PE microenvironment can release factors able in supporting a preferential recruitment or expansion of CD56^bright^CD16^−^ NK cells, preventing either the maturation or cytotoxic function of NK cells. The PE-NK cells are not anergic or exhausted as they respond well to IL-2 *in vitro* [30], and even healthy donor NK cells are strongly conditioned by the PE. We found a significant decrease of perforin-positive PE-NK cells in both inflammatory and malignant conditions, with MFI data confirming these findings. In analyzing cytotoxic activity of NK cells using the CD107a degranulation assay against the K562 tumor cell target, the killing capacity is significantly diminished in both PB-NK and PE-NK cells. These are in contrast with the previous data concerning freshly isolated malignant PB-NK and PE-NK cells [30] using the same degranulation assay (performing 4 h incubation of NK cells with K562 target cells) [29]. However, they also observed lower concentrations of perforin and granzyme B in PE-NK cells as compared to PB-NK cells [30]. Also, our metastatic clinical samples differ from these previous reports, since ours comprise more heterogeneous tumor-type compositions, (lung adenocarcinomas, melanoma, renal, ovarian, hepatocellular, and pancreatic carcinoma) and did not include colon, gastric, bladder, and uterus carcinoma (Table 1).

NK cells isolated from inflammatory PE exhibit features very similar to those of NK cells of the two types of malignant PE analyzed; thus, the factors influencing NK cell polarization might be derived from inflammatory cells. PE also have numerous macrophages, when isolated from malignant pleural effusions, exhibit weak cytotoxic activity against tumor cells, and increase proliferative activity and ability to protect tumor cells from apoptosis [52]. It has been shown that the percentage of CD163^+^ macrophages in malignant PEs was higher than that found in nonmalignant PEs. CD163^+^ macrophages in malignant PE patients are a prognostic factor for progression-free survival, and M2-related cytokines were more expressed in malignant PE-derived CD163^+^ than in CD163^−^ macrophages [53]. In addition, CD163^+^CD14^+^ macrophages could be used as an immune diagnostic marker for malignant PE patients [54]. Pleural effusions from patients with mesothelioma induced an M2-like phenotype that repressed T cell activity [55]. Macrophages in malignant PE produce high levels of TGF*β* [56], which plays an important role in impaired T cell cytotoxicity and in *in vitro* treatment with anti-TGF*β* mAb restored the impaired T cell cytotoxic activity. The amount of TGF*β* is higher in malignant PE than in nonmalignant PE [57] both of which are substantially higher than plasma TGF*β*. Treatment of healthy NK cells with TGF*β* induces a dNK-like phenotype [25, 58–60], including CD49a expression and production of angiogenic factors.

NK cells from patients with NSCLC were predominantly CD56^bright^CD16^−^, and we show that they are endowed with proangiogenic features similar to dNK cells [25]. PE-NK cells display an increased expression of CD49a decidua NK surface marker and were poorly mature (low expression of CD57) and yet activated (high expression CD69), all of which are characteristics of dNK cells. PE-NK cells exhibit a slight decrease of NKp30 (NCR3) and an enhancement of NKG2A. We found that PE-NK cells display a higher amount of intracellular VEGF as compared to autologous and healthy PB-NK cells (Figure 7) that became statistically significant from those isolated from tmPE fluids. NK cells from tmPE were also able to support capillary-like formation, *in vitro* (Figure 8), suggesting an imbalance in the angiostatic versus proangiogenic factors in PE-NK cells.

The presence of partially activated yet poorly mature NK cells can be interpreted in a twofold manner: (1) they are cells that have not completed the maturation process and consequently are only partially activated, or (2) they may be fully differentiated NK cells that are dedifferentiated or blocked as a result of stimulation within the PE. From a clinical and therapeutic point of view, it is crucial to know the capacity of tumor NK cells to be rescued in their antitumor function by using activating cytokines such as IL-2. Although patient PB-NK and PE-NK cells are not fully anergic as they respond to stimulation by IL-2 in the culture medium, however, addition of TGF*β* or iPE or tmPE fluids resulted in a strong inhibition of this crucial effector function of NK cells, even in healthy donor NK cells, suggesting a predominant role for the PE tumor microenvironment in the establishment of an alternative NK cell activation status. Other cytokines than IL-2 could be assessed in a clinical point of view, such as IL-15, IL-12, and IL-18, or combined cytokine treatments, knowing that these associations are associated with severe cytokine toxicity *in vivo*, and some are currently in clinical trials [61, 62]. However, some cytokines, for example, IL-15, induce both signal transducer and activator of transcription (STAT) 5, a cytokine favoring NK maturation and an antiangiogenic state [63], and STAT3, which blocks cytotoxicity [64], depending upon the context. These immunostimulatory cytokines could also be employed as adjuvants in conjunction with other treatments to boost and augment the efficacy of the antitumor response, for example, tumor-targeting vaccines, immune checkpoint blockade, or adoptive preactivated NK cell infusion [65, 66].

## 5. Conclusions

Our data suggest a crucial role for PE tumor microenvironment in shaping NK cell polarization from killer to builder proangiogenic cells resembling decidual NK cells, placing NK cells as new inflammatory hallmark for malignant pleural effusions.

## Figures and Tables

**(a):** 

Healthy controls (h)			
*n* = 14 (M: *n* = 8; F: *n* = 6)			
	Gender	Age	Diagnosis
1	M	45	Healthy subject
2	M	61	Healthy subject
3	M	57	Healthy subject
4	M	57	Healthy subject
5	M	65	Healthy subject
6	M	46	Healthy subject
7	M	76	Healthy subject
8	M	46	Healthy subject
9	F	47	Healthy subject
10	F	59	Healthy subject
11	F	65	Healthy subject
12	F	70	Healthy subject
13	F	58	Healthy subject
14	F	62	Healthy subject

Age: mean + SEM = 58.1 + 3.4.

**(b):** 

Inflammatory pleural effusion (iPE)			
*n* = 18 (M: *n* = 16; F: *n* = 2)			
	Gender	Age	Diagnosis
1	M	64	Acute pleurisy
2	F	74	Chronic pleurisy
3	M	45	Chronic pleurisy
4	M	72	Chronic pleurisy
5	M	74	Chronic pleurisy
6	M	80	Chronic pleurisy
7	M	80	Chronic pleurisy
8	M	83	Chronic pleurisy
9	M	85	Chronic pleurisy
10	M	59	Inflammatory pleural effusion
11	M	72	Inflammatory pleural effusion
12	M	77	Inflammatory pleural effusion
13	F	75	Pachypleuritis
14	M	65	Pachypleuritis
15	M	69	Pachypleuritis
16	M	71	Pachypleuritis
17	M	75	Pachypleuritis
18	M	79	Pachypleuritis

Age: mean + SEM = 72.2 + 2.2.

**(c):** 

Malignant primary tumor pleural effusion (ptPE)			
*n* = 18 (M: *n* = 9; F: *n* = 9)			
	Gender	Age	Diagnosis
1	F	70	Epithelioid and sarcomatoid mesothelioma
2	M	66	Epithelioid and sarcomatoid mesothelioma
3	F	66	Epithelioid mesothelioma
4	F	77	Epithelioid mesothelioma
5	F	82	Epithelioid mesothelioma
6	M	49	Epithelioid mesothelioma
7	M	52	Epithelioid mesothelioma
8	M	73	Epithelioid mesothelioma
9	M	73	Epithelioid mesothelioma
10	M	80	Epithelioid mesothelioma
11	F	80	Epithelioid mesothelioma
12	F	90	Mesothelioma
13	M	67	Mesothelioma
14	F	72	Mesothelioma
15	F	79	Mesothelioma
16	F	85	Mesothelioma
17	M	70	Mesothelioma
18	M	79	Mesothelioma

Age: mean + SEM = 72.8 + 2.5.

**(d):** 

Malignant tumor metastasis pleural effusion (tmPE)			
*n* = 27 (M: *n* = 11; F: *n* = 16)			
	Gender	Age	Diagnosis
1	F	53	Hepatocellular carcinoma
2	F	74	Ovarian carcinoma
3	M	82	Lung squamous cell carcinoma
4	F	52	Breast cancer
5	F	64	Breast cancer
6	F	59	Unknown cell carcinoma
7	F	73	Melanoma
8	M	88	Neuroendocrine carcinoma
9	F	68	Pancreatic cancer
10	F	49	Lung adenocarcinoma
11	F	50	Lung adenocarcinoma
12	F	67	Lung adenocarcinoma
13	F	68	Lung adenocarcinoma
14	F	69	Lung adenocarcinoma
15	F	75	Lung adenocarcinoma
16	F	79	Lung adenocarcinoma
17	F	88	Lung adenocarcinoma
18	F	91	Lung adenocarcinoma
19	M	46	Lung adenocarcinoma
20	M	58	Lung adenocarcinoma
21	M	64	Lung adenocarcinoma
22	M	70	Lung adenocarcinoma
23	M	75	Lung adenocarcinoma
24	M	77	Lung adenocarcinoma
25	M	78	Lung adenocarcinoma
26	M	79	Lung adenocarcinoma
27	M	58	Renal carcinoma

Age: mean + SEM = 68.7 + 2.4.

## Abbreviations

dNK: Decidual NK cells
FACS: Fluorescence-activated cell sorting
FBS: Foetal bovin serum
FGF1: Fibroblast growth factor 1
FGF2: Fibroblast growth factor 2
HBV: Hepatitis B virus
HIV: Human immunodeficiency virus
HUVEC: Human umbilical vein endothelial cell
ILC: Innate lymphoid cells
iPE: Inflammatory pleural effusion
MFI: Mean fluorescence intensity
NK: Natural killer
NSCLC: Non-small-cell lung cancer
PB: Peripheral blood
PE: Pleural effusion
PGE: Prostaglandin E
PGLYRP2: Peptidoglycan recognition protein 2
PlGF: Placental growth factor
ptPE: Primary tumor pleural effusion
SDF-1: Stromal cell-derived factor-1
SFM: Serum-free medium
SN: Serum-free conditioned supernatants
TINK: Tumor-infiltrating NK cells
TANK: Tumor-associated NK cells
TGF*β*: Transforming growth factor-b
tmPE: Tumor metastatic pleural effusion
VEGF: Vascular endothelial growth factor.

## Supplementary Materials

Supplementary Materials Supplemental Figure 1: evaluation of the CD9, activatory, and inhibitory receptor expression on total NK cell. Flow cytometric analysis on NK cells in samples from healthy individuals (hPB), peripheral blood (iPB) and pleural effusion (iPE) from patients with inflammatory disease, peripheral blood (ptPB) and pleural effusion (ptPE) from patients with primary tumor, and peripheral blood (tmPB) and pleural effusion (tmPE) from patients with tumor metastasis showed no difference in CD9 expression levels in terms of positive cell percentage (A) and mean fluorescence intensity (MFI) (B). We observed a downregulation of NKp30 surface receptor (C) and upregulation of the NKG2A inhibitory receptor (D) in all PE samples as compared to the PB-NK cells. The NKG2D activatory receptor (E) was relatively unchanged. Data are shown as mean ± SEM of 18 samples. Supplemental Figure 2: CD49a-decidual marker expression on total NK cells. CD49a expression was significantly increased in NK cells from pleural effusion (particularly in ptPE and in tmPE) as compared to PB-NK cell samples (A, B). Representative dot plots of CD49a distribution in healthy donors and patients with inflammatory, primary, and metastatic tumor PE are shown, respectively (C). Data are shown as mean ± SEM of 39 samples; ^∗^ *p* < 0.05, ^∗∗^ *p* < 0.01, and ^∗∗∗^ *p* < 0.001 (*p* values are shown). Supplemental Figure 3: CD57 and CD69 expression on total NK cells. Flow cytometric analysis on NK cells from healthy individuals (hPB), peripheral blood (iPB) and pleural effusion (iPE) from patients with inflammatory disease, peripheral blood (ptPB) and pleural effusion (ptPE) from patients with primary tumor, and peripheral blood (tmPB) and pleural effusion (tmPE) from patients with tumor metastasis revealed a decrease percentage of mature NK cells correlated to the downregulation of CD57 marker in PE samples as compared with PB and healthy donors (A). The upregulation of CD69, an activating and decidual marker, was observed in PE samples compared to autologous and healthy control PB-NK cells (C). Representative dot plots of CD57 (B) and CD69 (D) distribution in healthy donors and patients with inflammatory, primary, and metastatic tumor PE are shown, respectively. Data are shown as mean ± SEM of 39 samples; ^∗^ *p* < 0.05, ^∗∗^ *p* < 0.01, and ^∗∗∗^ *p* < 0.001 (*p* values are shown). Supplemental Figure 4: perforin secretion by total NK cells. Flow cytometric analysis showed a downregulation of perforin^+^ total NK cells in iPE and ptPE in term of percentage of positive cells (A) and mean intensity of fluorescence (MFI) (B). Representative dot plots of perforin expression in healthy donors and patients with inflammatory, primary, and metastatic tumor PE are shown, respectively (C). Data are shown as mean ± SEM of 34 samples; ^∗^ *p* < 0.05, ^∗∗^ *p* < 0.01, ^∗∗∗^ *p* < 0.001 (*p* values are shown). Supplemental Figure 5: VEGF production by total NK cells. Flow cytometric analysis on NK cells from healthy individuals (hPB), peripheral blood (iPB) and pleural effusion (iPE) from patients with inflammatory disease, peripheral blood (ptPB) and pleural effusion (ptPE) from patients with primary tumor, and peripheral blood (tmPB) and pleural effusion (tmPE) from patients with tumor metastasis revealed an increased production of VEGF in PE samples as compared with PB and healthy donors (A). Representative dot plots of VEGF production by healthy donors and patients with inflammatory, primary, and metastatic tumor PE are shown, respectively (B). Data are shown as mean ± SEM of 34 samples; ^∗^ *p* < 0.05 and ^∗∗^ *p* < 0.01 (*p* values are shown).
