# Supplementary figures and images for: Natural Killer Cells from Malignant Pleural Effusion Are Endowed with a Decidual-Like Proangiogenic Polarization
Source: J Immunol Res. 2018 Mar 29;2018:2438598. doi: 10.1155/2018/2438598 (PMC5896269; doi:10.1155/2018/2438598)

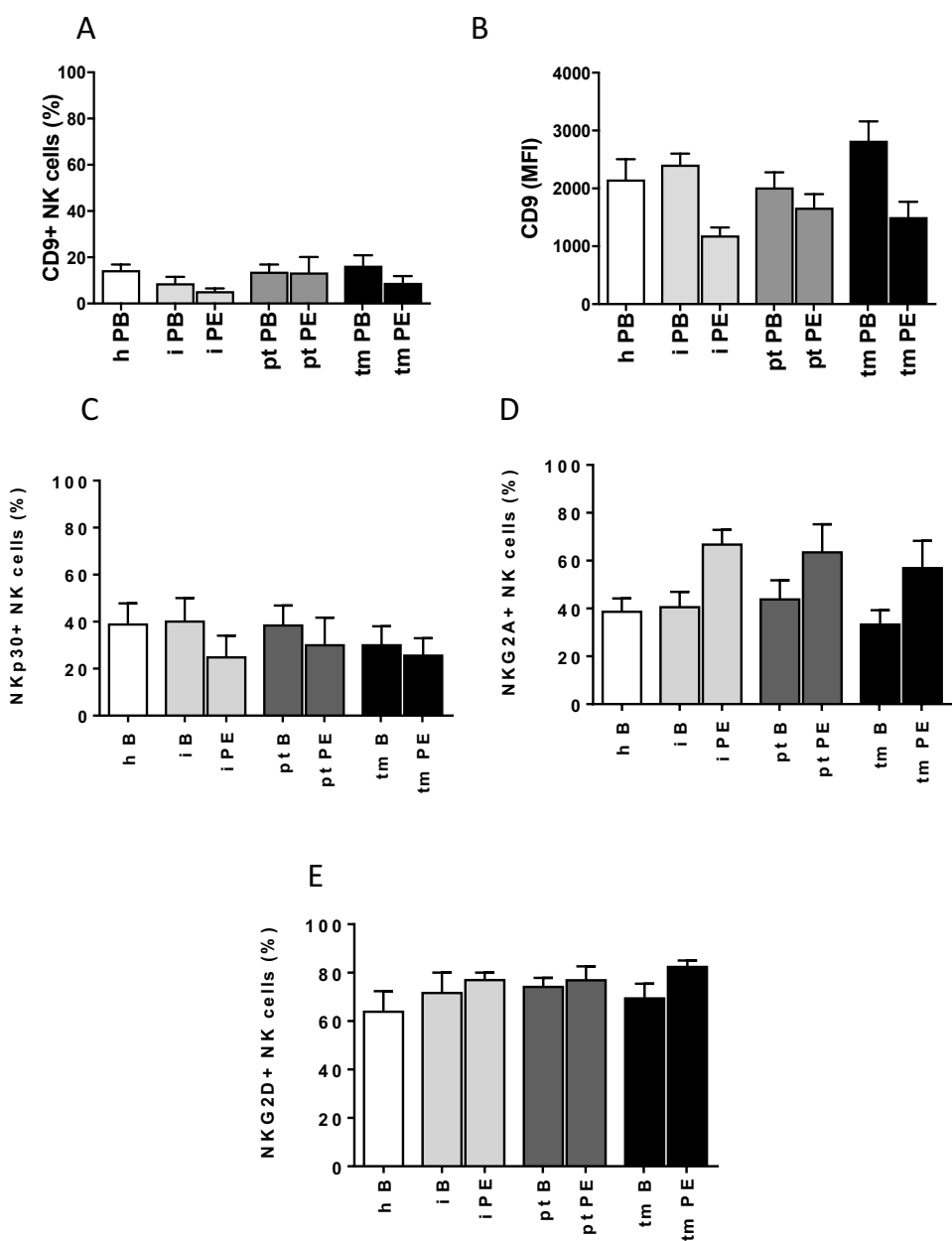

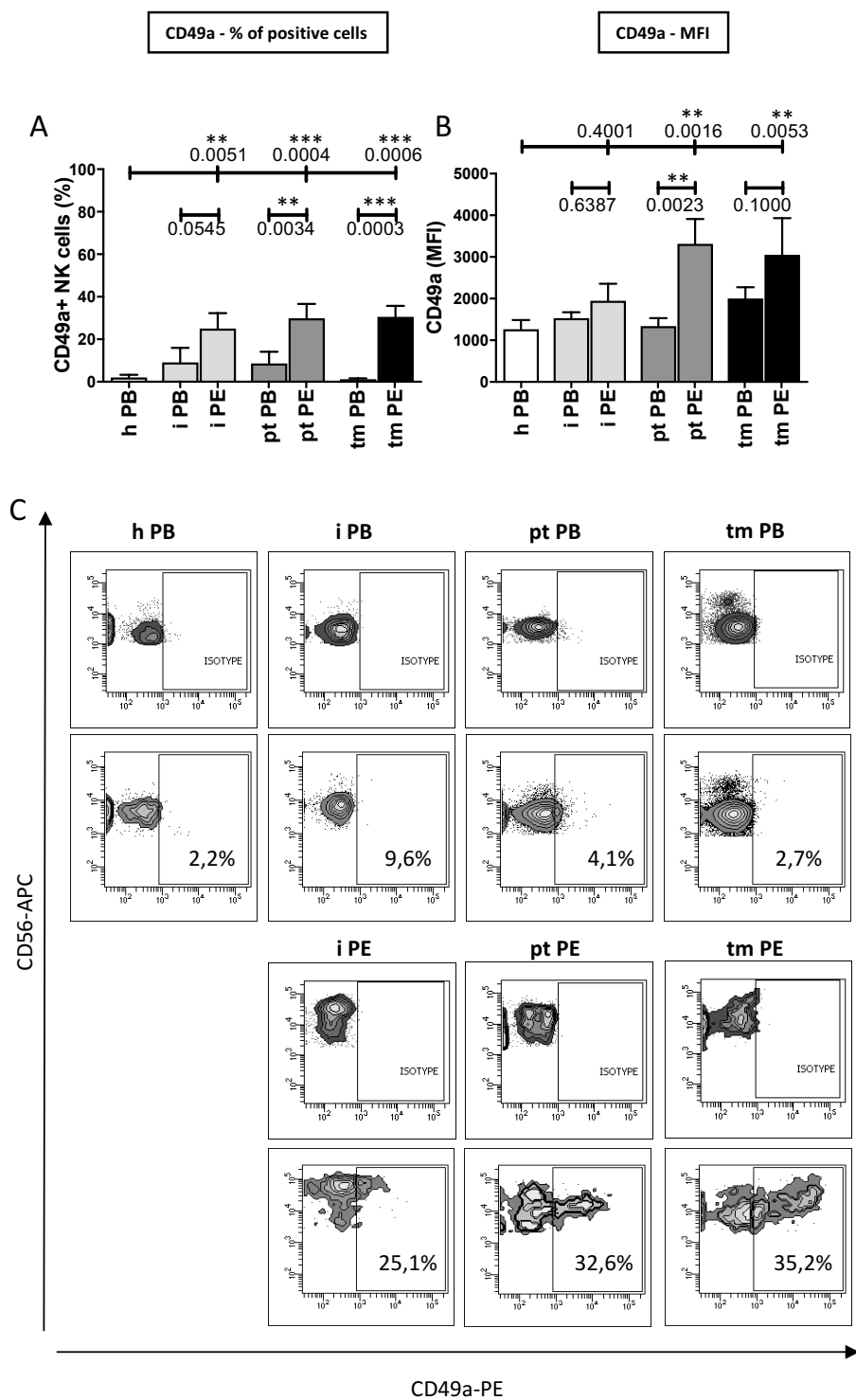

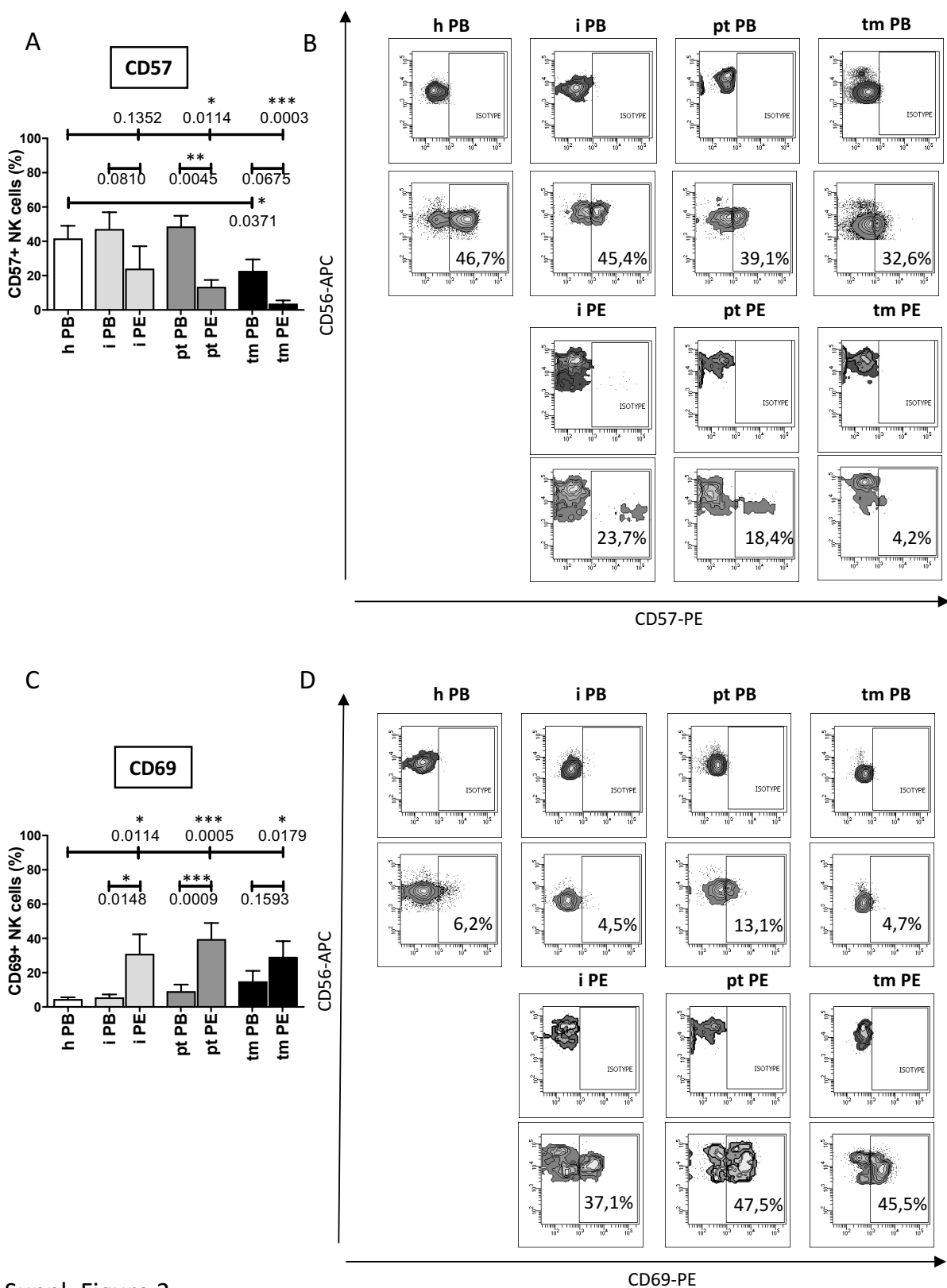

Suppl. Figure 3

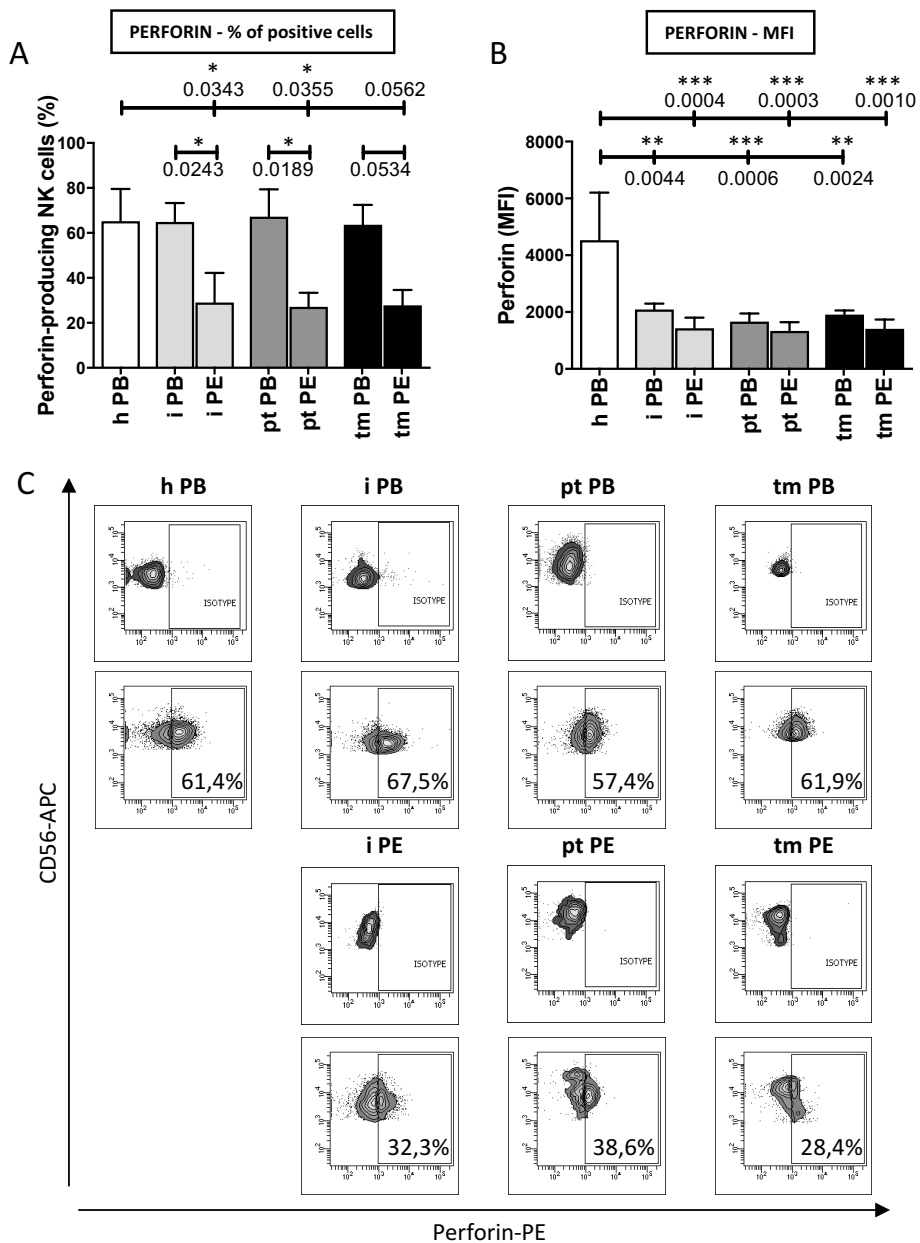

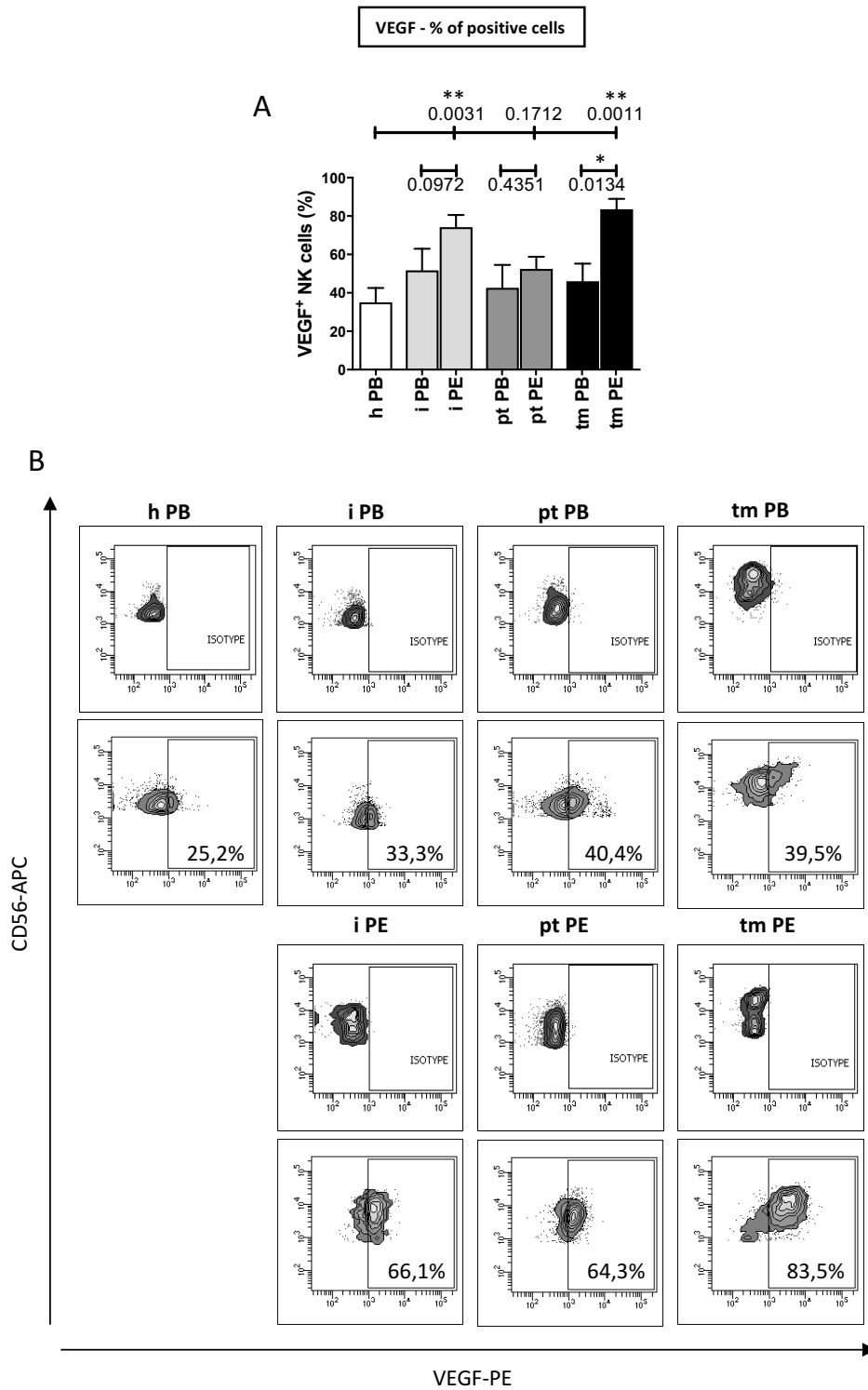

Suppl. Figure 5

Supplement: Supplementary Materials — Supplemental Figure 1: evaluation of the CD9, activatory, and inhibitory receptor expression on total NK cell. Flow cytometric analysis on NK cells in samples from healthy individuals (hPB), peripheral blood (iPB) and pleural effusion (iPE) from patients with inflammatory disease, peripheral blood (ptPB) and pleural effusion (ptPE) from patients with primary tumor, and peripheral blood (tmPB) and pleural effusion (tmPE) from patients with tumor metastasis showed no difference in CD9 expression levels in terms of positive cell percentage (A) and mean fluorescence intensity (MFI) (B). We observed a downregulation of NKp30 surface receptor (C) and upregulation of the NKG2A inhibitory receptor (D) in all PE samples as compared to the PB-NK cells. The NKG2D activatory receptor (E) was relatively unchanged. Data are shown as mean ± SEM of 18 samples. Supplemental Figure 2: CD49a-decidual marker expression on total NK cells. CD49a expression was significantly increased in NK cells from pleural effusion (particularly in ptPE and in tmPE) as compared to PB-NK cell samples (A, B). Representative dot plots of CD49a distribution in healthy donors and patients with inflammatory, primary, and metastatic tumor PE are shown, respectively (C). Data are shown as mean ± SEM of 39 samples; ∗ p < 0.05, ∗∗ p < 0.01, and ∗∗∗ p < 0.001 (p values are shown). Supplemental Figure 3: CD57 and CD69 expression on total NK cells. Flow cytometric analysis on NK cells from healthy individuals (hPB), peripheral blood (iPB) and pleural effusion (iPE) from patients with inflammatory disease, peripheral blood (ptPB) and pleural effusion (ptPE) from patients with primary tumor, and peripheral blood (tmPB) and pleural effusion (tmPE) from patients with tumor metastasis revealed a decrease percentage of mature NK cells correlated to the downregulation of CD57 marker in PE samples as compared with PB and healthy donors (A). The upregulation of CD69, an activating and decidual marker, was observed in PE sam [file 2438598.f1.pdf]
